# *De Novo* Characterization of a *Cephalotaxus hainanensis* Transcriptome and Genes Related to Paclitaxel Biosynthesis

**DOI:** 10.1371/journal.pone.0106900

**Published:** 2014-09-09

**Authors:** Fei Qiao, Hanqing Cong, Xuefei Jiang, Rongxiang Wang, Junmei Yin, Dan Qian, Zhunian Wang, Peter Nick

**Affiliations:** 1 Key Laboratory of Crop Gene Resources and Germplasm Enhancement in Southern China, Ministry of Agriculture/Tropical Crops Genetic Resources Institute, Chinese Academy of Tropical Agricultural Sciences, Danzhou, Hainan, China; 2 Key Laboratory of Protection and Development Utilization of Tropical Crop Germplasm Resources (Hainan University), Ministry of Education/College of Horticulture and Landscape Architecture, Hainan University, Haikou, Hainan, China; 3 Molecular Cell Biology, Botanical Institute, Karlsruhe Institute of Technology, Karlsruhe, Germany; Technische Universität Dresden, Medical Faculty, Germany

## Abstract

*Cephalotaxus hainanensis*, an endangered plant, is known to contain several metabolites with anti-cancer activity. Despite its clinical impact, the alkaloid metabolism of this species has remained largely uncharacterized. The potential of *Cephalotaxus* for metabolic engineering of medically interesting compounds has, so far, not been exploited, due to the almost complete lack of molecular information. We have therefore performed a high throughput RNA-seq analysis and assembled the transcriptome *de novo*. Raw reads comprising 4.3 Gbp were assembled *de novo* into 39,416 unique sequences (unigenes) with a mean length of 1,089.8 bp and a total assembly size of 45.8 Mbp, which equals to more than 50 times the number of *Cephalotaxaceae* sequences currently deposited in the GenBank (as of August 2013). As proof of principle for medically interesting pathways, gene fragments related to paclitaxel biosynthesis were searched and detected. To verify their functionality, the metabolic product paclitaxel, and its precursor baccatin III, were identified in the leaves of *C. hainanensis* by HPLC, and shown to be induced by MeJA. This finding demonstrates exemplarily the potential of the annotated transcriptome as information resource for the biotechnological exploitation of plant secondary metabolism.

## Introduction

Plants generate around 10^6^ specific secondary metabolites [1]. Most of these metabolites modulate the interaction of plants with other organisms and therefore many of them are pharmaceutically active. Often, those compounds are medically relevant, but cannot be synthetized technically and therefore have to be extracted and purified from their natural source. The underlying metabolic pathways are complex and often require the interaction of different cell types until the active compound is stored in specialized tissues [2], often even in specialized secretory cells. This renders extraction cumbersome, inefficient and costly. Very often, the respective plants are endangered. For instance, the Pacific Yew was brought to the verge of extinction by the discovery that paclitaxel can block the growth of human tumors. Biotechnological approaches, for instance based on tissue culture, would be an alternative. Especially in a situation, where small quantities of a highly priced product have to be produced, Green Molecular Farming turns out to excel other systems of biotechnological production such as transgenic animals or microorganisms [3]. The focus of Green Molecular Farming has been on the production of recombinant proteins, whereas the wealth of plant secondary compounds has remained mostly unexploited. One limitation is our still incomplete knowledge on the underlying pathways and the genes driving metabolic complexity. For two decades, the impressive advances of molecular biology have remained confined to few model systems – in the plant field mostly thale cress and rice – whereas medically interesting species were not accessible. The technological breakthroughs in high-throughput technologies, especially next-generation sequencing, have now allowed extending molecular analysis to more “exotic” models that so far had remained out of scope.

*Cephalotaxus*, the sole representative of the family Cephalotaxaceae, is distributed through southern and eastern Asia. As a member of this genus, *Cephalotaxus hainanensis* is endemic to the tropical island of Hainan. *C. hainanensis* is an evergreen conifer tree which can reach 20–25 m in height. Due to its slow growth and over-exploitation for timber and medical purposes, *Cephalotaxus hainanensis* is already classified as an endangered plant in China.

Alkaloids of *Cephalotaxus* were reported back to 1954 [4]. Some 50 alkaloids, falling into two types (cephalotaxine-type and homoerythrine-type) have been isolated and identified from this genus so far [5]. Among these alkaloids, drupangtonine, 11α-hydroxy-homodeoxy-harringtonine, 11β-hydroxy-homodeoxy-harringtonine, 11β-hydroxy-deoxy-harringtonine [6], neoharringtonine, homo-neoharringtonine, (3′S)-hydroxyl-neoharringtonine [7], nordeoxy-harringtonine, homodeoxy-harringtonine and bishomodeoxy-harringtonine have attracted medical interest [8], due to their cytotoxicity against P-388 leukemia cells. Most recently, homoharringtonine-based induction therapies were shown to be more effective against acute myeloid leukemia compared to daunorubicin and cytarabine, hitherto considered as the gold standard for induction chemotherapy [9]. Moreover, some *Cephalotaxus* alkaloids harbor also activities against epidermoid carcinoma [10], lymphoma [11], and nasopharynx carcinoma [12], as well as against pertinent human parasites such as *Plasmodium falciparum* and *Leishmania major* [13].

Although it is principally possible to synthetize these rare alkaloids and their analogues chemically [14], this approach has not been pursued intensively, probably due to low efficiency. Biosynthesis should be possible, and a pathway has been already proposed by Powell [15]. However, sequence information on the enzymes driving the biosynthesis of these alkaloids has remained elusive. The advent of RNA-Seq technique has facilitated large-scale gene discovery [16], and in fact, with this method, a gene cluster involved synthesis of an anticancer alkaloid in poppy has been identified [17].

In the current study, we apply this strategy to *Cephalotaxus hainanensis*, as case study for a pharmacologically interesting species, for which only limited sequence information is available. Illumina RNA-Seq technology was used to generate 51,442,422 short reads containing a total of 5,195,684,622 nucleotide bases. By *de-novo* assembly, 39,416 unigenes with an average length of 1090 bp were identified by combining these reads and annotated with respect to their potential function. This sequence information will be used as a platform for global gene discovery in *Cephalotaxus*. To demonstrate the feasibility of this strategy, sequences related to paclitaxel biosynthesis were identified using this platform, and the metabolites predicted by the presence of these genes, paclitaxel and its precursor baccatin III, subsequently could be detected in leaves of *C. hainanensis*.

## Materials and Methods

### Preparation of plant samples and RNA isolation

Seedlings of *C. hainanensis* were collected in the greenhouse of the Institute of Tropical Crop Genetic Resources, Chinese Academy of Tropical Agricultural Sciences (CATAS) originating from Jianfengling in Ledong county, Hainan province, China. The leaves were harvested and frozen immediately in liquid nitrogen. Total RNA was extracted using a CTAB-based isolation procedure [18], and purified with the Axyprep multisource total RNA miniprep kit (Axygen Scientific, Inc., Hangzhou, China). The quantity and quality of the isolated total RNA was verified using UV spectrophotometry (DU800, Beckman Coulter, USA) and gel electrophoresis, respectively.

### Library construction and Illumina sequencing

A *C. hainanensis* leaf transcriptome library was constructed using an mRNA-seq assay for paired-end Illumina sequencing, which was performed at Majorbio Biopharm Technology Co., Ltd. (Shanghai, China). Poly(A) mRNA was isolated from total RNA by using Sera-mag Magnetic Oligo (dT) Beads (Thermo Fisher Scientific, USA), and then mRNA-enriched RNAs were chemically fragmented to short pieces using the RNA Fragmentation Reagent (Ambion, USA). Double-stranded cDNA was synthesized using the SuperScript Double-Stranded cDNA Synthesis Kit (Invitrogen, Carlsbad, CA). Subsequently, the Illumina Paired End Sample Prep kit (Illumina, USA) was used to construct a RNA-seq library which then was sequenced by Illumina HiSeq 2000 (Illumina, San Diego, CA).

### *De novo* Assembly

Due to the absence of reference genomic sequences, *de-novo* assembly was applied to construct transcripts from these RNA-seq reads. Briefly, the raw reads generated by Illumina Hiseq 2000 were initially processed to generate clean reads by removing the adapter sequences and low quality bases at the 3′ end. Then, the transcriptome was assembled *de novo* using the short-read assembly program Trinity (http://trinityrnaseq.sourceforge.net/) following a published method [19]. Clean reads with a specified length of overlap were firstly combined to form longer contiguous sequences (contigs), and then these reads were mapped back onto the contigs using pair-end joining and gap-filling *in silico*. A minimum of three read pairs was used as criterion to define order and distance between two contigs to exclude chimeric reads arising from miss-assembly. This strategy allowed to detect contigs originating from the same transcript and also to calculate the distances between the contigs. Subsequently, the reconstructed contigs were assembled further to obtain longer sequences. This procedure was reiterated until the constructed sequence could not be extended on either end using the Trinity software. Such saturated combined contigs were considered as unique transcripts. Finally, these unique assembled transcripts were further processed using the sequence-splicing redundancy removal routine of Trinity to yield non-redundant transcripts defined as unigenes.

### ORF Prediction and Annotation of Gene Functions

All operationally defined *C. hainanensis* unigenes were analyzed for open reading frames using the Trinity software. In parallel, these sequences were subjected to similarity search in the NCBI non-redundant (Nr) protein database (http://www.ncbi.nlm.nih.gov/), the String database (http://string-db.org/), and the Swiss-Prot database (http://www.expasy.ch/sprot) using Blastx with an E-value of less than 1e^−5^. Unigene sequences that did not produce hits in the first database were reiterated by a search in the next database. Based on the Blast results, the unigene sequences were translated into the related peptide sequences. Protein sequences of high similarity to these peptide sequences were retrieved from these databases along with their functional annotations to infer functions from the information available for homologous genes.

Based on the annotation in the non-redundant (Nr) protein database, the Blast2GO program (http://www.blast2go.com/b2ghome) was used to search for gene ontological annotations according to molecular function, biological process and cellular component for the *C. hainanensis* unigenes [20]. After retrieving the GO annotations for every unigene, they were classified using the WEGO software (http://wego.genomics.org.cn/cgi-bin/wego/index.pl) with respect to functional classification, which allows to define functional clusters [21]. In parallel, the unigenes were also aligned to the Clusters of Orthologous Groups (COG) database (http://www.ncbi.nlm.nih.gov/COG/) to predict and classify possible functions. Pathway assignments were carried out according to the Kyoto Encyclopedia of Genes and Genomes (KEGG) pathway database (http://www.genome.jp/kegg) using Blastx with E-value threshold of 10^−5^.

### EST-SSR detection and primer design

The 32,687 unigenes were screened for potential SSR markers using the msatcommander (http://code.google.com/p/msatcommander/) following previously described methods [22]. The parameters were adjusted for identification of perfect mono-nucleotide, di-nucleotide, tri-nucleotide, tetra-nucleotide, penta-nucleotide, and hexa-nucleotide motifs with a minimum of 10, 6, 4, 4, 4, and 4 repeats, respectively. Mononucleotide repeats were ignored since distinguishing genuine mononucleotide repeats from polyadenylation products and single nucleotide stretch errors generated by the sequencing itself turned out to be difficult. Primer pairs were designed using BatchPrimer3 software [23]. The major parameters for primer pair design were set as follows: primer length of 18–23 bases (optimal 20 bases), PCR product size of 100–400 bp (optimally 200 bp), GC content of 40–70% (optimally 50%), and annealing temperatures of 50–60°C (optimally 55°C).

### HPLC analysis

Leaves of *C. hainanensis* were ground thoroughly with mortar and pestle in liquid nitrogen. 0.25 g of leaf powder were transferred to 1.25 ml methanol and homogenized by an ultrasonic processor for 30 min. The homogenate was incubated for 1 h in the dark at room temperature, then mixed vigorously by (vortex), and centrifuged at 13000 rpm for 10 min. Then the supernatant was filtrated through a 0.22 µm syringe filter (Life Sciences) prior to injection into the HPLC.

Paclitaxel and baccatin III were analyzed using a high performance liquid chromatograph, HPLC (Hitachi, 2130 series, Japan) equipped with an octadecylsilyl column (Kromasil 100-5C_18_, 4.6 mm×25 cm, particle size 5 µm), a UV detector, and a quaternary valve. The flow rate was 1.0 ml⋅min^−1^, and the injection volume was 20 µl. The mobile phases included acetonitrile (ACN), formic acid and water in the following gradient: 30 min water/ACN/formic acid (58/40/2, v/v); 3 min water/ACN/formic acid (8/90/2, v/v); 5 min water/ACN/formic acid (58/40/2, v/v). Paclitaxel and baccatin III were quantified and identified using an external standard on the basis of retention time and UV-VIS spectra. The standards for paclitaxel and baccatin III (Aladdin, Shanghai) were dissolved in methanol at a concentration of 1 mg⋅ml^−1^, respectively. Calibration curves determined using these standards were linear (r^2^≥0.999) and used for quantification of the samples. At least three independent experimental series were conducted.

To observe, whether paclitaxel and baccatin III contents can be enhanced by elicitation, the leaves were rubbed with a solution of 1 µM MeJA and harvested at indicated time points (0.5, 12, 24, 36, 48, 72 h) for HPLC analysis.

## Results

### Illumina sequencing and *de novo* assembly

**Figure 1:**
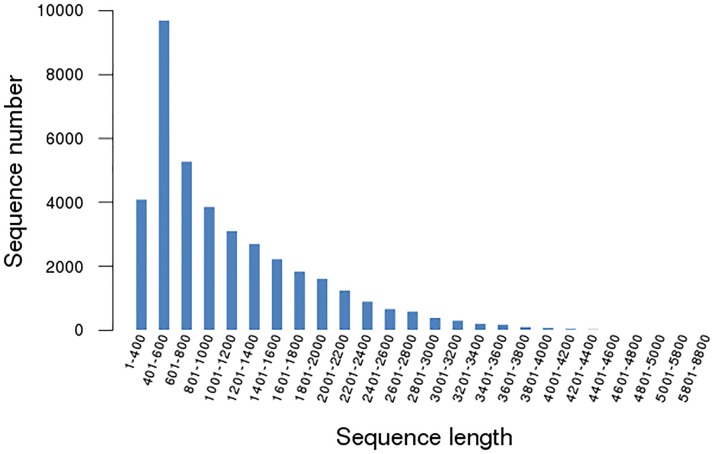
Distribution of frequency (absolute numbers) over the length. The constructed unigenes from *de-novo* assembly of *Cephalotaxus hainanensis* short reads were generated by Illumina Hiseq 2000 sequencing. The total number of assembled unigenes is 39,416 unigene.

The raw data from the *C. hainanensis* experimental samples yielded a total of 51,442,422 short sequence reads consisting of 5,195,684,622 nucleotides (nt) total, with an average length of 90 bp for each short read (NCBI SRA accession No.: SRR1509462). In total, 39,416 unigenes could be constructed. This Transcriptome Shotgun Assembly project has been deposited at DDBJ/EMBL/GenBank under the accession GBHQ00000000. The version described in this paper is the first version, GBHQ01000000. As shown in Figure 1, the sequence length of these assembled unigenes ranged from 351 bp to 8,604 bp, with an average length of 1,089.78 bp. The number of unigenes decreased with increasing unigene length (Figure 1). The quantitative parameters of the assembled transcriptome are summarized in Table 1.

**Table 1:** Quantitative characteristics of the reconstructed *C. hainanensis* transcriptome.

Type	Number
Total genes (including ORF)	25,731
Total unigenes	39,416
Total residues (bp)	42,954,608
Average length per unigene	1,089.78
Largest unigene (bp)	8,604
Smallest unigene (bp)	351

### Gene prediction and annotation of predicted proteins

By ORF analysis, 30,107 unigene sequences harboring a Coding Sequence (CDS) could be identified. The residual 9,309 unigenes did not produce hits in the ORF analysis. All 30,107 CDS could be translated into peptide sequences and all 39,416 unigenes with or without ORF were annotated by blast alignment with different protein and nucleotide databases (for details refer to Table S1 and Table S2).

### GO classification and KEGG pathway analysis

**Figure 2:**
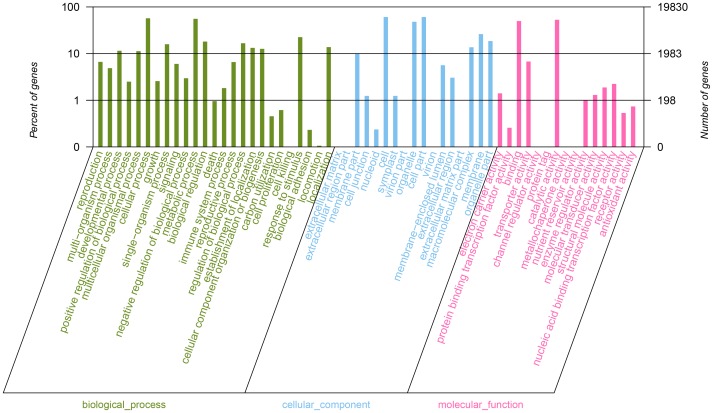
Frequency distribution ontological classifications. The results are grouped into three main categories: biological process, cellular component and molecular function. The right y-axis indicates the number of genes in a category. The left y-axis indicates the percentage of a specific category of genes in that main category. Note that this axis is logarithmic to appropriately depict the large variations in abundance.

To classify the predicted functions of the constructed *C. hainanensis* unigenes, the Blast2GO and the Kyoto Encyclopedia of Genes and Genomes (KEGG) pathway databases were utilized. Based on sequence homology, a total of 19,830 (50.31%) sequences could be categorized into 57 functional groups (Table S3). Most of the GO terms of unigenes fell into the category biological process (56,479 terms or 43.54%), 49,577 terms (or 38.22%) into the group cellular component, and 23,671 terms (18.25%) into the group molecular function. Among the sub classifications, cellular process (11,325 unigenes or 28.73%), cell part (12,069 unigenes, or 30.62%), and catalytic activity (10541 unigenes, or 26.74%) were dominant, respectively (Figure 2). The high number of unigenes putatively involved in cellular (11,325) and metabolic (11,040) processes indicate that the source tissue was metabolically very active (Figure 2).

To identify the biological pathways that were active in the source tissue of *C. hainanensis*, the 39,416 annotated sequences were mapped to the reference pathways in the KEGG database [24]. In total, 9,355 unigene sequences could be assigned to 290 KEGG pathways. The dominating pathways were classified as metabolic pathways (2,307 unigenes), biosynthesis of secondary metabolites (1,082 unigenes), microbial metabolism in diverse environments (398 unigenes), and spliceosome (351 unigenes). We also specifically investigated the assembled unigenes with respect to paclitaxel synthesis, and we could identify 72 unigenes with a putative role for the terpenoid backbone biosynthesis. These annotations provided a valuable resource to investigate specific processes, functions, and pathways linked with paclitaxel synthesis in *C. hainanensis* (some representatives are shown in Table 2, see also Table S4).

**Table 2:** List of selected unigenes involved in paclitaxel biosynthesis.

Gene ID	Annotation from homology in GenBank	Similarity (%)	E-value
1782	geranylgeranyl diphosphate synthase [*Taxus canadensis*]	93%	0
2312	taxane 10-beta-hydroxylase (5-alpha-taxadienol-10-beta-hydroxylase; cytochrome P450 725A1) [*Taxus wallichiana var. chinensis*]	81%	0
3857	geranylgeranyl diphosphate synthase 6 [*Picea abies*]	91%	8e-131
5165	phenylalanine aminomutase [*Taxus wallichiana var. chinensis*]	86%	0
7490	taxadiene 5-alpha hydroxylase [*Taxus cuspidata*]	79%	0
8229	geranylgeranyl diphosphate synthase 5 [*Picea abies*]	84%	0
13420	taxadiene synthase (Taxa-4(5),11(12)-diene synthase) [*Taxus brevifolia*]	78%	1e-167
13597	taxadiene synthase (Taxa-4(5),11(12)-diene synthase) [*Taxus baccata*]	82%	0
13862	taxadiene 5-alpha hydroxylase [*Taxus wallichiana var. chinensis*]	86%	2e-112
16282	13-alpha-hydroxylase [*Taxus wallichiana var. chinensis*]	79%	4e-165
16379	13-alpha-hydroxylase [*Taxus wallichiana var. chinensis*]	81%	2e-119

### Frequency and distribution of EST-SSRs

In total, 4,489 sequences containing 5,314 SSRs were identified from the 39,416 consensus sequences, with 644 of these EST sequences containing more than one SSR (Table S5). The EST-SSR frequency in the *C. hainanensis* transcriptome was 11.39%, and the distribution density was 1,652.23 per Mb. The most abundant type of repeat motif were tri-nucleotides (2,719, or 51.16%), followed by mono-nucleotides (1,936, or 36.43%), whereas di-nucleotides, hexa-nucleotides, tetra-nucleotides (83, or 1.66%), and penta-nucleotides were rare (Table 3). SSRs with four tandem repeats were the most common (Table 4), followed by five, and six tandem repeats. The dominant repeat motives were AGG/CCT (453, or 15.36%), AAG/CTT (447, or 15.16%), and GAT/GCT (414, or 14.04%) as shown in Table 4. In contrast, not a single CG/CG repeat was discovered in the entire database. Based on these parameters, 3,806 primer pairs were designed that are now available to screen Cephalotaxaceae germplasm for polymorphisms (Table S6).

**Table 3:** Frequency of single-stranded repeats (SSR) in the ESTs constructed from *C. hainanensis*.

Motif length	Repeat numbers								Total	%
	4	5	6	7	8	9	10	>10		
Mono	-	-	-	-	-	-	891	1045	**1,936**	**36.43**
Di	-	-	195	67	49	31	25	32	**399**	**7.51**
Tri	1861	544	202	96	13	2	-	1	**2,719**	**51.16**
Tetra	70	13	-	-	-	-	-	-	**83**	**1.66**
Penta	41	14	-	-	-	-	-	-	**55**	**1.04**
Hexa	98	11	4	5	1	1	-	2	**122**	**2.28**
**Total**	**2,070**	**582**	**401**	**168**	**63**	**34**	**916**	**1,080**	**5,314**	
**%**	**38.95**	**10.95**	**7.54**	**3.16**	**1.19**	**0.64**	**17.24**	**20.32**		

**Table 4:** Frequency of individual di- and trinucleotide SSR motives in the ESTs constructed from *C. hainanensis*.

Motif length	Repeat numbers								Total	%
	4	5	6	7	8	9	10	>10		
AC/GT	-	-	25	10	5	2	-	5	**47**	**1.59**
AG/CT	-	-	59	22	8	7	4	4	**104**	**3.53**
AT/AT	-	-	111	35	36	22	21	23	**248**	**8.41**
CG/CG	-	-	-	-	-	-	-	-	**0**	**0**
AAC/GTT	122	14	19	1		-	-	-	**156**	**5.29**
AAG/CTT	237	147	38	23	1	-	-	1	**447**	**15.16**
AAT/ATT	187	44	24	15	1	1	-	-	**272**	**9.22**
ACC/GGT	141	32	6	6	4	-	-	-	**189**	**6.41**
ACG/CTG	19	2	2	-	-	-	-	-	**23**	**0.78**
ACT/ATG	13	-	-	-	-	-	-	-	**13**	**0.44**
AGC/CGT	209	64	30	2	1	-	-	-	**306**	**10.38**
AGG/CCT	285	103	35	28	2	-	-	-	**453**	**15.36**
AGT/ATC	132	20	4	4	-	-	-	-	**160**	**5.43**
CCG/CGG	58	32	14	10	2	1	-	-	**117**	**3.97**
GAT/GCT	289	86	30	7	2	-	-	-	**414**	**14.04**
**Total**	**1,692**	**544**	**397**	**163**	**62**	**33**	**25**	**33**	**2,949**	**1.59**
%	**57.38**	**18.45**	**13.46**	**5.53**	**2.10**	**1.12**	**0.85**	**1.12**		

### Prediction and experimental verification of the paclitaxel pathway in *Cephalotaxus*

To verify exemplarily the quality and potential of the newly constructed molecular resource, sequences potentially related to paclitaxel biosynthesis were searched and compiled specifically. Data from this study were compared with sequences from the String data base, and as shown in Table 2, a number of putative homologues to genes known to act in paclitaxel biosynthesis could be identified, including taxadiene synthase, geranyl geranyl diphosphate synthase (GGPPs), phenylalanine aminomutase (PAM), taxane 2-alpha hydroxylase, taxane 7-beta hydroxylase, taxane 5-alpha hydroxylase, taxane 10-beta hydroxylase, taxane 13-alpha hydroxylase. The presence of these sequences predicted that paclitaxel biosynthesis is functional also in *Cephalotaxus*.

**Figure 3:**
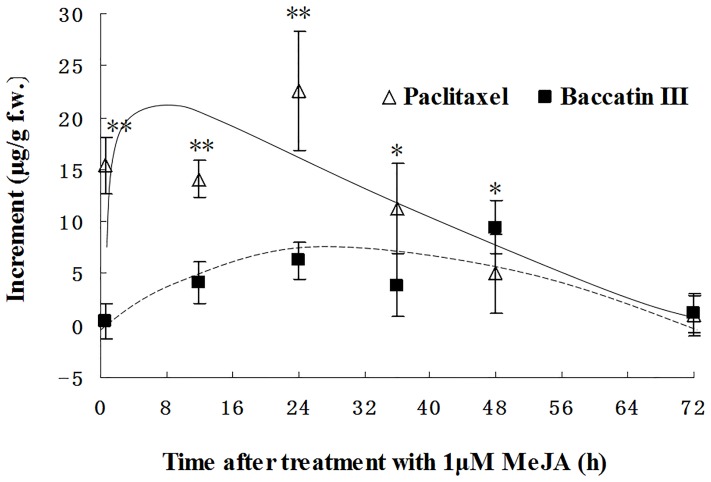
Time course of paclitaxel and baccatin III accumulation in leaves of *C. hainanensis* after induction with 1 µM MeJA. Mean values and standard errors from three independent experimental series are shown. * Significantly different from the untreated control at the 95% confidence level, ** significantly different form the untreated control at the 99% confidence level.

To verify this prediction experimentally, the presence of paclitaxel and its precursor baccatin III in extracts from *C. hainanensis* was investigated by HPLC. In fact, 24.35 µg of paclitaxel per g fresh weight (corresponding to 0.0096% dry weight), and 17.81 µg of baccatin III per g fresh weight (corresponding to 0.004% dry weight) could be detected in leaf extracts from *C. hainanensis* respectively. Thus, paclitaxel content in this species is comparable to that in the bark of *Taxus brevifolia* (with a dry-weight content of 0.004–0.01% paclitaxel) [25], which up to 1993 was the predominant source for paclitaxel. By elicitation with 1 µM of MeJA, the content of paclitaxel can be stimulated rapidly and significantly (*P<0.001*) by almost 60% compared to the ground level (Figure 3). Interestingly, the paclitaxel precursor, baccatin III shows a more or less constant steady state level, which means that the stimulated conversion into paclitaxel is compensated by an equivalent formation of baccatin III. The induction of paclitaxel accumulation is transient with a maximum at around 1 d after elicitation and subsequent return to the ground level.

## Discussion

We used high-throughput RNA sequencing to construct the transcriptome of *Cephalotaxus hainanensis*, an endangered gymnosperm species, for which very little molecular data are available. By this approach we could expand the sequence information available for the Cephalotaxaceae by more than 50 times as compared to the information available in public databases. As a proof-of-principle to verify quality and usefulness of this novel molecular resource, we could predict the presence of the paclitaxel pathway in *C. hainanensis* and subsequently verified experimentally the presence of paclitaxel and its precursor baccatin III in leaf extracts from this species.

Paclitaxel was first found in Pacific Yew and other *Taxus* species. Later, endophytic fungi of *Taxus* species were also found to contain paclitaxel [26], [27]. Although more than ten of the genes involved in paclitaxel biosynthesis have been cloned and identified, it is still unrealistic to produce it by molecular farming using recombinant microbes, since the information on the genes relevant for this pathway has remained sketchy [28]. Currently, paclitaxel is produced commercially by plant cell culture (www.phytonbiotech.com). The original approach, to use leaves and twigs of different species of Yews as source, has brought some of these slowly growing trees to the verge of extinction.

The transcriptome database for *C. hainanensis* provides novel tools for the biotechnological use of an organism which contains multiple interesting metabolites with anticancer activity. By combining the strategy to generate compounds by plant cell culture (which has been successful for the case of *Taxus*), it should also be possible to produce both paclitaxel and other *Cephalotaxus* alkaloids with medical potential in cell cultures of *C. hainanensis*. Even a partial insight into interesting genes of the pathway would allow searching conditions, by which the production of these compounds can be boosted. Our findings that methyl jasmonate can induce paclitaxel accumulation [29], and also promotes the dynamic equilibrium of its precursor baccatin III indicate that the entire pathway can be activated by jasmonate signalling. Since the induction of paclitaxel, and the concomitant changes in the dynamic equilibrium of baccatin III are already observed at the first measured time point (30 min after induction with MeJA), they are unlikely to be caused by gene activation, but rather must be caused by posttranslational regulation of enzymatic activities or substrate availability. Since jasmonate signalling has been investigated intensively, it is now possible to identify the respective molecular players from *Cephalotaxus* and to identify the bottlenecks that constrain the accumulation of these valuable alkaloids. Careful analysis of the promoter motives responsible for these bottlenecks might be used to generate elite cell strains with elevated alkaloid synthesis.

One issue that has received little attention is, why plant synthetize these metabolites with antitumor activity. This question is highly relevant for biotechnological applications, because it is linked with the so far modest yields obtained for paclitaxel. The fact that paclitaxel content can be induced in response to both abiotic and biotic signals, favors this hypothesis [30], [31]. However, the target of paclitaxel is beta tubulin [32], a fairly conserved molecule. In fact, the beta tubulin from *C. hainanensis* shows almost 95% similarity on the amino acid level, when compared to tubulins from other species (data not shown). So, the paclitaxel produced by these plants is expected to bind their native tubulin and to affect the function of microtubules, which may be one reason for the extremely slow growth of these trees and the constrained synthesis of this product. Based on the molecular resource generated in the current work, it is possible to search for signals that could be used as efficient inducers of the pathway, which would allow tailoring metabolic activity for the needs of bio fermentation.

## Conclusions

The wealth of plant secondary metabolism, so far, has only been partially exploited for biotechnological applications. One of the reasons for this drawback has been the limited availability of molecular information that over many years has been confined to few canonical model organisms such as thale cress or rice. Unfortunately, this small group of canonical model plants does not cover those species that are of interest with respect to their pharmacological potential. The advent of next-generation sequencing technology has made it feasible to elucidate genomes and transcriptomes also for these non-canonical systems. The current study adopts this strategy and provides the first comprehensive sequencing and functional annotation for the rare, but medically interesting species *C. hainanensis*. As exemplarily shown for paclitaxel synthesis, this molecular resource can be used to identify the molecular players responsible for the synthesis of valuable compounds. In the next step, the regulatory features of these molecular players will be characterized to identify constraints and bottlenecks. By genetic engineering and/or manipulation of cellular signaling, these constraints can be removed or controlled to give rise to cellular systems that allow for the synthesis of these compounds in an inducible manner as further step towards sustainable molecular farming.

## Supporting Information

Table S1 **Annotation of predicted proteins from** ***C. hainanensis*** **transcriptome.**
(XLS)

Table S2 **Genes predicted from** ***C. hainanensis*** **transcriptome.**
(XLS)

Table S3 **GO classification of all unigenes in** ***C. hainanensis*** **transcriptome.**
(XLS)

Table S4 **KEGG pathway analysis of all unigenes in** ***C. hainanensis*** **transcriptome.**
(XLS)

Table S5 **Prediction and analysis of EST-SSRs in** ***C. hainanensis*** **.**
(XLS)

Table S6 **Designed EST-SSRs primers for** ***C. hainanensis*** **.**
(XLS)
